# Melatonin Combined with Endoplasmic Reticulum Stress Induces Cell Death via the PI3K/Akt/mTOR Pathway in B16F10 Melanoma Cells

**DOI:** 10.1371/journal.pone.0092627

**Published:** 2014-03-19

**Authors:** Han Sung Kim, Tack-Joong Kim, Yeong-Min Yoo

**Affiliations:** 1 Department of Biomedical Engineering, College of Health Science, Yonsei University, Wonju, Gangwon-do, Republic of Korea; 2 Division of Biological Science and Technology, College of Science and Technology, Yonsei University, Wonju, Gangwon-do, Republic of Korea; UAE University, Faculty of Medicine & Health Sciences, United Arab Emirates

## Abstract

This study investigated B16F10 melanoma cell death induced by melatonin combined with endoplasmic reticulum (ER) stress through the PI3K/Akt/mTOR pathway. Cell viability was significantly decreased after treatment with melatonin combined with ER stress from thapsigargin or tunicamycin compared to no treatment or treatment with melatonin only. Combined melatonin and ER stress also significantly reduced expression of p85β, p-Akt (Ser473, Thr308), and p-mTOR (Ser2448, Ser2481) compared to treatment with melatonin only. The ER stress protein p-PERK and p-eIF2α were significantly increased under combined melatonin and ER stress treatment compared to no treatment or treatment with melatonin only. Combined melatonin and ER stress significantly reduced Bcl-2 protein and augmented Bax protein compared to melatonin-only treatment. Also, the combined treatment significantly lowered expression of catalase, Cu/Zn-SOD, and Mn-SOD proteins compared to melatonin only. Expression of p85β was significantly more decreased under treatment with melatonin and thapsigargin or tunicamycin plus the PI3K inhibitors LY294002 or wortmannin than under treatment with only melatonin or a PI3K inhibitor. The PI3K downstream target p-Akt (Ser473, Thr308) showed significantly decreased expression under treatment with melatonin and thapsigargin or tunicamycin plus PI3K inhibitors than under treatment with melatonin or PI3K inhibitors only. These results indicate that survival of B16F10 melanoma cells after combined treatment with melatonin and ER stress inducers is suppressed through regulation of the PI3K/Akt/mTOR pathway. Melatonin combined with thapsigargin or tunicamycin appears to be a promising strategy for effective melanoma treatment.

## Introduction

Melanomas are malignant tumors that arise from melanocytes, which produce black or brown melanin pigment in skin, but are also found in other parts of the body such as the bowel and the eye. Melanocytes synthesize melanin from tyrosine to protect the body from damaging ultraviolet radiation [1]. Melanocytes are found in various areas of the body including the skin, bowel, and eyes but are predominantly located in the epidermis; over 90% of all melanomas are cutaneous [1]–[3]. Melanoma is the fifth most common cancer in the United States, causing up to 75% of deaths related to skin cancer [4]. Melanoma is curable if detected early; however, metastatic melanoma requires continued therapy [5].

Melatonin has direct anticancer or anti-apoptotic effects on different types of human tumors [6]–[11] and functions as a broad-spectrum antioxidant [12]–[18]. Melatonin is also reported to induce apoptotic or autophagic cell death [19]–[21]. Numerous studies have demonstrated that melatonin has anti-proliferative effects in melanoma cells [22]–[29], and that these effects are related to the cell line-specific response of melatonin-binding receptors [25]–[29].

The phosphatidyl inositol 3-kinase/mammalian target of rapamycin (PI3K/mTOR) pathway is an important target for therapies for numerous cancers such as lung carcinoma, thyroid carcinoma, breast cancer, gastrointestinal carcinoma, and bladder carcinoma [30]–[33]. PI3K and its downstream targets AKT/PKB and mTOR are central in physiological processes such as cell growth, survival, motility, differentiation, and proliferation, and in the development of malignant disease [30]. The PI3K family is divided into three classes [34]. Class IA contains the PI3K proteins that are important for regulating proliferation and tumorigenesis. Class IA PI3Ks are heterodimeric molecules composed of a p110 catalytic subunit and a p85 regulatory subunit. The PI3K/Akt/mTOR pathway is frequently activated in human melanoma and is a possible therapeutic target for melanoma treatment [35]–[38].

Zha et al. [39] demonstrate that melatonin sensitizes human hepatoma cells to endoplasmic reticulum (ER) stress-induced cell death. In this study, we found that combined melatonin and ER stress treatment induced cell death in B16F10 melanoma cells through the PI3K/Akt/mTOR pathway. This finding suggests that targeting PI3K/Akt/mTOR could be an effective strategy for the melanoma therapy.

## Materials and Methods

### Cell Culture

B16F10 cells were obtained from Korea Cell Line Bank (Seoul, Korea) and were cultured in Dulbecco’s modified Eagle’s medium (DMEM, GibcoBRL, Gaithersburg, MD, USA) supplemented with 5% heat-inactivated fetal bovine serum (FBS, GibcoBRL) at 37°C with 5% CO_2_ in a humidified incubator.

### ER Stress and Treatment of PI3k Inhibitors

B16F10 cells were cultured in DMEM medium plus 1% heat-inactivated FBS with or without melatonin (0.1, 0.5, 1 mM) and/or thapsigargin (1 μM) (Calbiochem, San Diego, MO, USA) for 6 hr, or with tunicamycin (5 μg/mL) (Calbiochem) for 16 hr in a 37°C and 5% CO_2_ incubator. Melatonin (Sigma, St Louis, MO, USA) was dissolved in dimethyl sulfoxide, and cells were treated with melatonin for 24 hr. To determine the effects of 20 μM LY294002 and 2 μM wortmannin (Calbiochem), cells were treated for 1 hr and co-exposed with or without melatonin and/or thapsigargin or tunicamycin.

### Cell Viability Assay

Cell survival was determined using a Cell Counting Kit-8 (Dojindo, Tokyo, Japan). Briefly, B16F10 cells were cultured in 96-well plates (Corning Inc., Corning, NY, USA) at 5×10^3^ cells per well with or without dissolved melatonin. After 24 hr, cells were washed and treated with the Cell Counting Kit-8, and the plate was incubated in the dark for 4 hr. Absorbance at 450 nm was read using a microplate reader (Epoch System, BioTek Instruments, Winooski, VT, USA). Percent viability was calculated as (absorbance of melatonin-treated cells/absorbance of control cells)×100.

### Western Blotting

Cells were harvested, washed twice with ice-cold PBS, and resuspended in 20 mM Tris-HCl buffer (pH 7.4) containing protease inhibitors (0.1 mM phenylmethylsulfonyl fluoride, 5 μg/mL aprotinin, 5 μg/mL pepstatin A, and 1 μg/mL chymostatin) and phosphatase inhibitors (5 mM Na_3_VO_4_ and 5 mM NaF). Whole cell lysates were prepared using 20 strokes of a Dounce homogenizer, followed by centrifugation at 13,000×*g* for 20 min at 4°C. Protein concentration was determined using the Bicinchoninic acid assay (BCA) (Sigma, St Louis, MO, USA). Proteins (40 μg) or media (20 μL) were separated by 12% sodium dodecyl sulfate-polyacrylamide gel electrophoresis (SDS-PAGE) and transferred onto polyvinylidene difluoride membranes. Membranes were incubated with antibodies against p-mTOR (Ser2448, Ser2481) and mTOR (1∶1000) (Cell Signaling Technology, Beverly, MA, USA**)**; p-Akt (Thr308, Ser473) and Akt (1∶1000) (Cell Signaling Technology); p85α and p85β (1∶500) (Santa Cruz Biotechnology, Santa Cruz, CA, USA); p-PERK and PERK (1∶1000) (Cell Signaling Technology); p-eIF2α and eIF2α (1∶1000 dilution) (Cell Signaling Technology); Bax and Bcl-2 (1∶500) (Santa Cruz Biotechnology); catalase (1∶500) (Santa Cruz Biotechnology); Cu/Zn-SOD (1∶500) (Santa Cruz Biotechnology); Mn-SOD (1∶500) (Santa Cruz Biotechnology) and actin (1∶1000) (Santa Cruz Biotechnology). Membranes were incubated with anti-rabbit or anti-mouse IgG-conjugated horseradish peroxidase secondary antibodies (Santa Cruz Biotechnology) and ECL Western blotting reagents (Pierce Biotechnology, Rockford, IL, USA). Immunoreactive proteins were visualized by exposure to X-ray film. Protein bands were visualized by image scanning, and optical density was measured using ImageJ analysis software (version 1.37; Wayne Rasband, NIH, Bethesda, MD, USA) after data were corrected by background subtraction and normalized to actin as an internal control.

### Statistical Analysis

Significant differences were detected by ANOVA, followed by Tukey’s test for multiple comparisons. Analysis was performed using Prism Graph Pad v4.0 (Graph Pad Software Inc., San Diego, CA, USA). Values are expressed as mean ± standard deviation (SD) of at least three separate experiments, with representative experiments shown in the figures. *P*<0.05 was considered statistically significant.

## Results

### Cell Survival in Thapsigargin or Tunicamycin Combined with Melatonin Treatment

**Figure 1:**
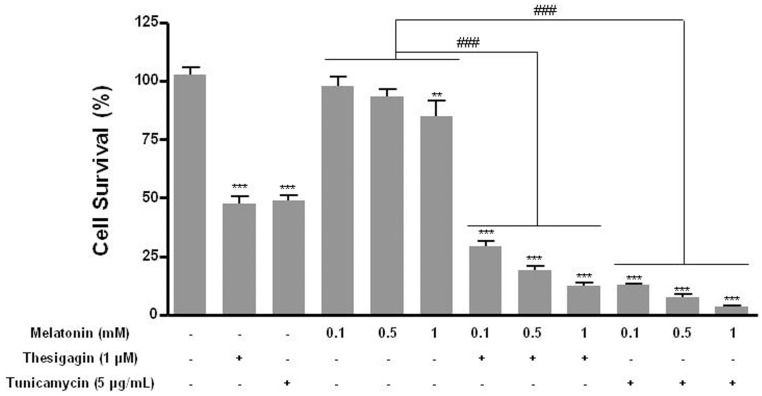
Cell viability of B16F10 cells after treatment with melatonin with or without thapsigargin or tunicamycin. Cell survival of B16F10 cells was determined after 24± SD of three experiments. *P<0.05; **P<0.01; ***P<0.001 vs. untreated control; ^###^ *P*<0.001, melatonin vs. melatonin with thapsigargin or tunicamycin.

We investigated whether melatonin combined with ER stress induced death in B16F10 melanoma cells. Cell survival was significantly reduced by about 48% in ER stress conditions of thapsigargin or tunicamycin treatment and by about 30–37% when ER stress induced by thapsigargin or tunicamycin was combined with melatonin treatment. Comparisons were to treat with or without melatonin only (Fig. 1).

### p85β, p-Akt, and p-mTOR in Thapsigargin or Tunicamycin Combined with Melatonin Treatment

**Figure 2:**
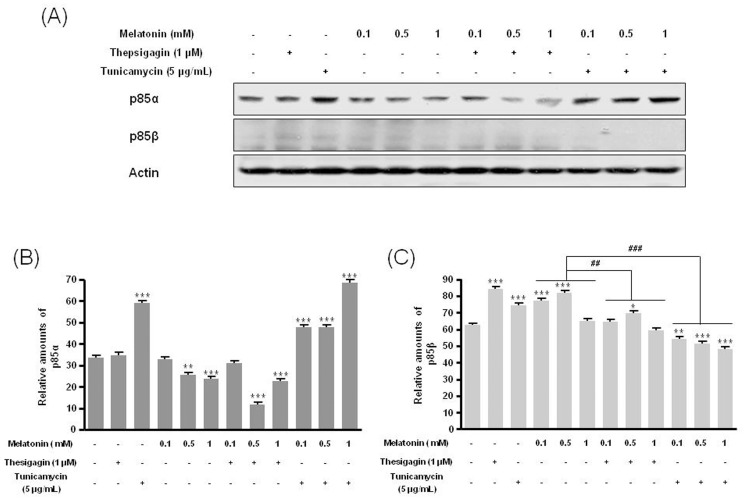
Expression of p85α and p85β in B16F10 cells after melatonin with or without thapsigargin or tunicamycin treatment. B16F10 cells were cultured with or without melatonin (0.1, 0.5, 1 mM) and/or thapsigargin (1 μM) for 6 hr or tunicamycin (5 μg/mL) for 16 hr. p85α and p85β expression was analyzed by Western blot (A). Relative amounts of p85α (B) and p85β (C) quantified as described in Materials and methods. Data are mean ± SD of three experiments. ***P<0.001 vs. untreated control; ^##^ *P*<0.01, ^###^ *P*<0.001, melatonin vs. melatonin with thapsigargin or tunicamycin.

**Figure 3:**
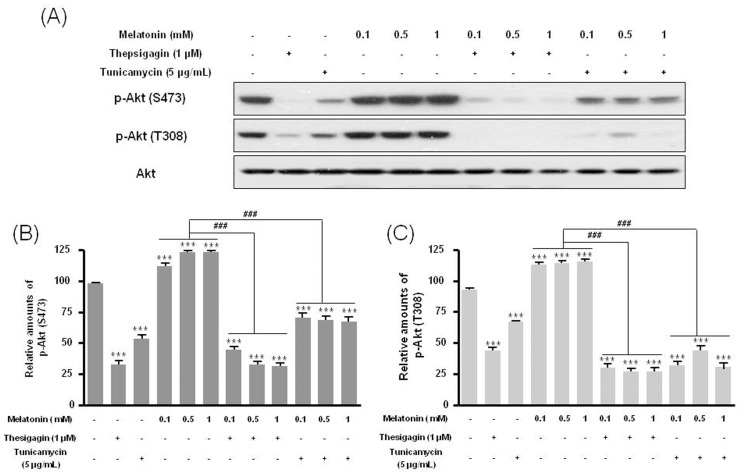
Phosphorylation of Akt (Ser473) and Akt (Thr308) in B16F10 cells after melatonin with or without thapsigargin or tunicamycin treatment. B16F10 cells were cultured with or without melatonin (0.1, 0.5, 1 mM) and/or thapsigargin (1 μM) for 6 hr or tunicamycin (5 μg/mL) for 16 hr. p-Akt (Ser473) and p-Akt (Thr308) were analyzed by Western blot (A). Relative amounts of p-Akt (Ser473) (B) and p-Akt (Thr308) (C) quantified as described in Materials and methods. Data are mean ± SD of three experiments. ***P<0.001 vs. untreated control; ^###^ *P*<0.001, melatonin vs. melatonin with thapsigargin or tunicamycin.

**Figure 4:**
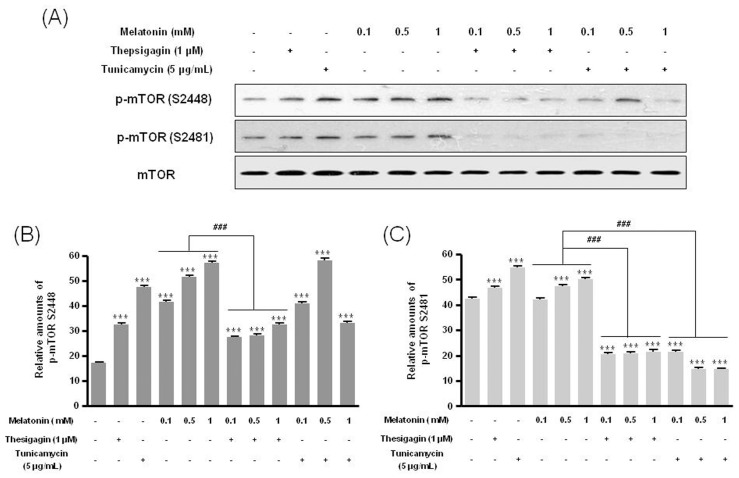
Phosphorylation of mTOR (Ser2448) and mTOR (Ser2481) in B16F10 cells after melatonin with or without thapsigargin or tunicamycin treatment. B16F10 cells were cultured with or without melatonin (0.1, 0.5, 1 mM) and/or thapsigargin (1 μM) for 6 hr or tunicamycin (5 μg/mL) for 16 hr. p-mTOR (Ser2448) and p-mTOR (Ser2481) were analyzed by Western blot (A). Relative amounts of p-mTOR (Ser2448) (B) and p-mTOR (Ser2481) (C) were quantified as described in Materials and methods. Data are mean ± SD of three experiments. ***P<0.001 vs. untreated control; ^###^ *P*<0.001, melatonin vs. melatonin with thapsigargin or tunicamycin.

Combined melatonin and ER stress significantly decreased p85β protein expression compared to melatonin-only treatment (Fig. 2). Under combined treatment, expression of the PI3K downstream proteins p-Akt (Ser473, Thr308) and p-mTOR (Ser2448, Ser2481) was significantly reduced compared to melatonin-only treatment (Figs. 3, 4).

### p-PERK and p-eIF2α in Thapsigargin or Tunicamycin Combined with Melatonin Treatment

**Figure 5:**
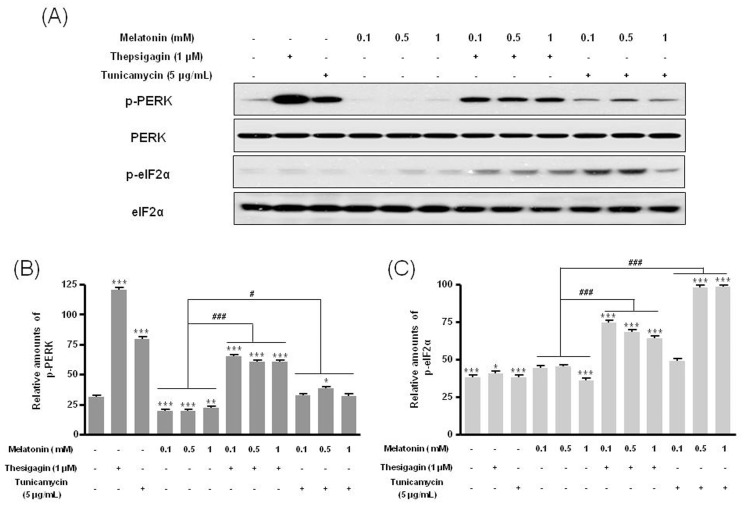
Phosphorylation of PERK and eIF2α proteins in B16F10 cells after melatonin with or without thapsigargin or tunicamycin treatment. B16F10 cells were cultured with or without melatonin (0.1, 0.5, 1 mM) and/or thapsigargin (1 μM) for 6 hr or tunicamycin (5 μg/mL) for 16 hr. p-PERK and p-eIF2α proteins were analyzed by Western blot (A). Relative amounts of p-PERK (B) and p-eIF2α (C) were quantified as described in Materials and methods. Data are mean ± SD of three experiments. ***P<0.001 vs. untreated control; ^#^ *P*<0.05, ^###^ *P*<0.001, melatonin vs. melatonin with thapsigargin or tunicamycin.

The ER stress protein p-PERK was significantly increased under treatment with melatonin and ER stress compared to treatment with or without melatonin (Figs. 5A, B). The protein p-eIF2α also significantly increased under combination treatment (Figs. 5A, C).

### Bcl-2/Bax, Catalase, Cu/Zn-SOD, and Mn-SOD in Thapsigargin or Tunicamycin Combined with Melatonin Treatment

**Figure 6:**
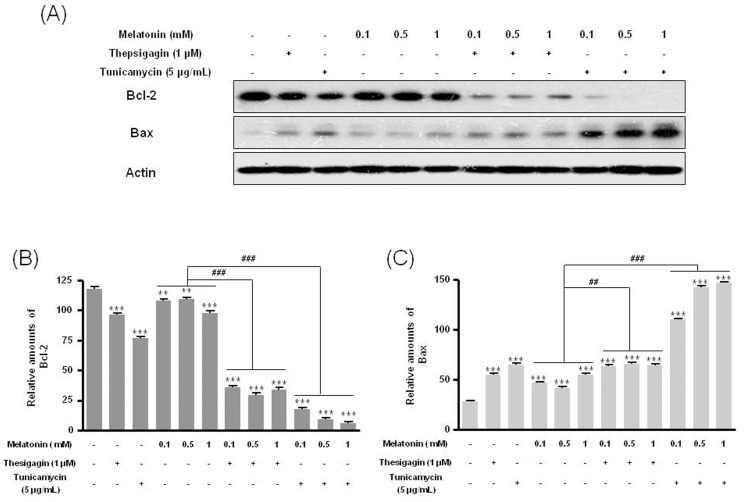
Expression of Bcl-2 and Bax in B16F10 cells after melatonin with or without thapsigargin or tunicamycin treatment. B16F10 cells were cultured with or without melatonin (0.1, 0.5, 1 mM) and/or thapsigargin (1 μM) for 6 hr or tunicamycin (5 μg/mL) for 16. Bcl-2 and Bax were analyzed by Western blot (A). Relative amounts of Bcl-2 (B) and Bax (C) were quantified as described in Materials and methods. Data are mean ± SD of three experiments. ***P<0.001 vs. untreated control; ^##^ *P*<0.01, ^###^ *P*<0.001, melatonin vs. melatonin with thapsigargin or tunicamycin.

**Figure 7:**
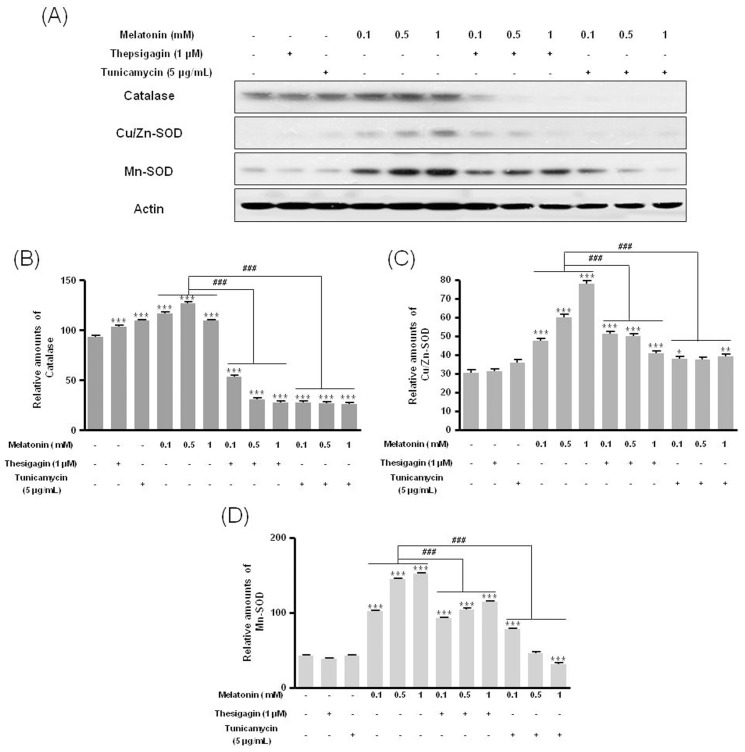
Expression of catalase, Cu/Zn-SOD, and Mn-SOD in B16F10 cells under melatonin with or without thapsigargin or tunicamycin treatment. B16F10 cells were cultured with or without melatonin (0.1, 0.5, 1 mM) and/or thapsigargin (1 μM) for 6 hr or tunicamycin (5 μg/mL) for 16 hr. Catalase, Cu/Zn-SOD, and Mn-SOD were analyzed by Western blot (A). Relative amounts of catalase (B), Cu/Zn-SOD (B), and Mn-SOD (D) quantified as described in Materials and methods. Data are mean ± SD of three experiments. ***P<0.001 vs. untreated control; ^###^ *P*<0.001, melatonin vs. melatonin with thapsigargin or tunicamycin.

Combined melatonin and ER stress significantly reduced Bcl-2 protein expression and augmented Bax expression compared to treatment with or without melatonin (Fig. 6). Also, the combined condition significantly decreased expression of catalase, Cu/Zn-SOD, and Mn-SOD proteins compared to melatonin only (Fig. 7).

### The PI3K Inhibitors LY294002 or Wortmannin Confirms the Expression of p85β Protein

**Figure 8:**
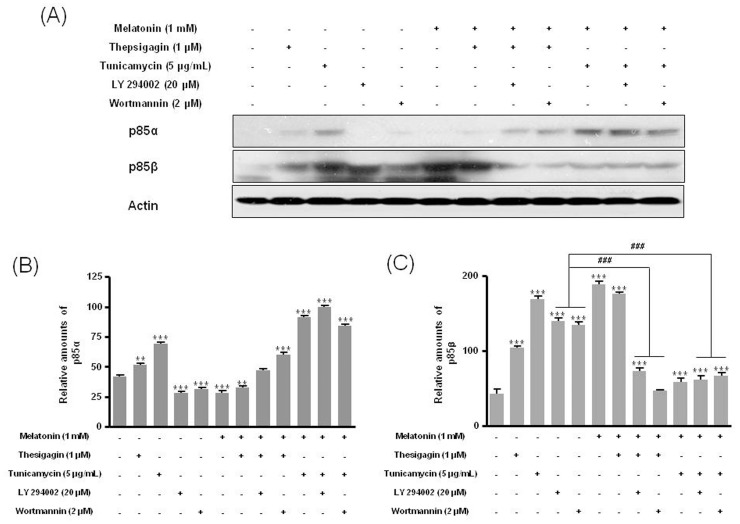
Expression of p85α, p85β, and p110 proteins in B16F10 cells after melatonin with or without thapsigargin/tunicamycin or LY 294002 or wortmannin treatment. B16F10 cells were cultured with or without melatonin (0.1, 0.5, 1 mM) and/or thapsigargin (1 μM) for 6 hr or tunicamycin (5 μg/mL) for 16 hr. Cells were treated with 20 μM LY 294002 or 2 μM wortmannin for 1 hr. Expression of p85α, p85β, and p110 was analyzed by Western blot (A). Relative amounts of p85α (B) and p85β (C) quantified as described in Materials and methods. Data are mean ± SD of three experiments. ***P<0.001 vs. untreated control; ^###^ *P*<0.001, LY294002 or wortmannin vs. melatonin with thapsigargin or tunicamycin or LY294002 or wortmannin.

**Figure 9:**
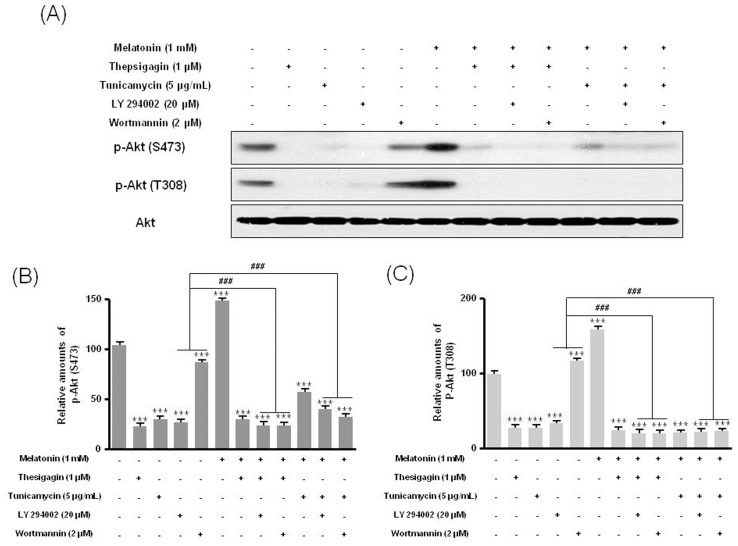
Phosphorylation of Akt (Ser473) and Akt (Thr308) in B16F10 cells under melatonin with or without thapsigargin or tunicamycin or LY294002 or wortmannin. B16F10 cells were cultured with or without melatonin (0.1, 0.5, 1 mM) and/or thapsigargin (1 μM) for 6 hr or tunicamycin (5 μg/mL) for 16 hr. Cells were treated with 20 μM LY294002 or 2 μM wortmannin for 1 hr. p-Akt (Ser473) and p-Akt (Thr308) were analyzed by Western blot (A). Relative amounts of p-Akt (Ser473) (B) and p-Akt (Thr308) (C) quantified as described in Materials and methods. Data are mean ± SD of three experiments. ***P<0.001 vs. untreated control; ^###^ *P*<0.001, LY294002 or wortmannin vs. melatonin with thapsigargin or tunicamycin or LY294002 or wortmannin.

Last, we found that the PI3K inhibitors LY294002 or wortmannin suppressed the expression of PI3K subunit p85β protein seen after combined melatonin and ER stress treatment of B16F10 melanoma cells. Expression of p85β decreased significantly after treatment with melatonin and thapsigargin or tunicamycin plus PI3K inhibitors than after treatment with only melatonin or PI3K inhibitors (Fig. 8). In addition, the PI3K downstream protein p-Akt (Ser473, Thr308) showed significantly lower expression after treatment with melatonin and thapsigargin or tunicamycin and PI3K inhibitors than after treatment with only melatonin or PI3K inhibitors (Fig. 9). These results indicate that survival of B16F10 melanoma cells after combined treatment with melatonin and ER stress inducers was suppressed through regulation of the PI3K/Akt/PKB-mTOR (p85β) pathway.

## Discussion

This study found that melatonin combined with thapsigargin or tunicamycin directly influenced cell death of B16F10 melanoma cells via the PI3K/Akt/mTOR pathway, suggesting that melatonin combined with ER stress could be a new therapeutic strategy for effective melanoma treatment.

Melatonin has an anti-proliferative function in melanoma cells with either a direct antitumor effect [22]–[25] or through melatonin receptors [26]–[29]. For direct anti-proliferation effects, the effective melatonin dose is 1 μM to 1 mM, and the effective time is 24 to 72 hr. For potential interactions with melatonin receptors, the dose is 10 pM to 0.1 μM for 72 hr in melanoma cells [22]–[29]. However, this pharmacological action of melatonin does not offer a promising strategy for preventing proliferation of melanoma cells. Instead, new strategies and potential alternative approaches using melatonin are needed for melanoma treatment. In this study, the PI3K/Akt/mTOR pathway in melanoma cells was demonstrated to be a new therapeutic target for melanoma treatment [30]–[38].

Sánchez-Hernández et al. [40] reported that the RAF inhibitor sorafenib induces apoptosis by dual targeting of BRAF and PI3K/AKT/mTOR signaling to effectively control melanoma disease. Marone et al. [41] used newly identified ATP-competitive PI3K/mTOR inhibitors to demonstrate that the PI3K and mTOR pathways inhibit tumor growth in a syngeneic B16 mouse melanoma tumor model. Furthermore, a combination of PI3K inhibitors and rapamycin has cooperative effects in other tumors *in vitro* [42]. Sinnberg et al. [43] studied combinations of the PI3K inhibitor LY294002 or the mTOR inhibitor rapamycin with the chemotherapeutics cisplatin or temozolomide, finding that these combinations significantly induced apoptosis of melanoma cells and completely suppressed invasive tumor growth of melanoma cells. These results indicate that the PI3K/mTOR signaling pathway effectively regulates melanoma disease and prevents tumor progression. In this study, we demonstrated that a combination of melatonin and thapsigargin or tunicamycin induced cell death through the PI3K/Akt/mTOR pathway in B16F10 melanoma cells. This treatment could be a new strategy for effective suppression of B16F10 melanoma cell proliferation.

In melanomas, the anti-apoptotic proteins Bcl-2, Bcl-xL, and Mcl-1 appear to increase while the pro-apoptotic protein Bax decreases with the progression of primary melanoma tumors and melanoma cells and might be involved in resistance to conventional therapies [44]–[46]. In our study, the combination of melatonin and thapsigargin or tunicamycin significantly downregulated Bcl-2 and upregulated Bax in melanoma cells. These and other studies suggest that the PI3K/AKT/mTOR signaling pathway modulates the expression of Bcl-2 and Bax family proteins [47]–[50]. Inhibition of mTOR with rapamycin- or mTOR-specific small interfering RNA downregulated Bcl-2 and Mcl-1 in anaplastic large-cell lymphoma cells [51]. Likewise, the RAF inhibitor sorafenib downregulates Bcl-2 and Mcl-1 through independent inhibition of mitogen-activated protein kinase (MAPK) [52]–[54]. Also, sorafenib together with rapamycin downmodulates Bcl-2 and Mcl-1 through mechanisms that are dependent on RAF and MAPK inhibition [46]. Therefore, Bcl-2 and Bax family proteins modulate melanoma proliferation through PI3K/mTOR or MAPK signaling pathways and could effectively regulate melanoma disease and prevent tumor progression.

The PI3K inhibitors LY294002 and wortmannin and the mTOR inhibitor rapamycin significantly augment growth inhibition by cisplatin and temozolomide. This results indicate a possible synergistic effect on melanoma cells treated with combinations of rapamycin or wortmannin and temozolomide [43]. Combinations of the PI3K inhibitors LY294002 and wortmannin or the mTOR inhibitor rapamycin with the chemotherapeutics cisplatin or temozolomide lead to a significant 2- to 3-fold increase in melanoma cell apoptosis, but MAPK pathway inhibitors do not significantly increase chemosensitivity [43]. Similarly, in our study, the PI3K inhibitors LY294002 or wortmannin in combination with melatonin and thapsigargin or tunicamycin induced cell death. We demonstrated that the PI3K inhibitors LY294002 and wortmannin in combination with melatonin and thapsigargin or tunicamycin influenced the expression of a PI3K subunit and p-Akt in B16F10 melanoma cells. PI3K inhibitors in combination with melatonin and thapsigargin or tunicamycin decreased expression of the PI3K subunit p85β and p110 proteins and the PI3K downstream molecule p-Akt (Ser473, Thr308) significantly more than treatment with melatonin or PI3K inhibitors only. These results indicate that the PI3K inhibitors LY294002 and wortmannin in combination with melatonin and thapsigargin or tunicamycin enhance the PI3K/AKT/mTOR signaling pathway.

Recent studies imply that intrinsic pathways for induction of cell death in cancer therapy are initiated by ER stress. Abnormal protein folding or calcium imbalance in the ER triggers ER stress and subsequently induces destruction responses in cells [55]–[58]. Cell death of human melanoma cells by ER stress induced to upregulate p-PERK and p-eIF2α could benefit the pharmaceutical development of anti-melanoma drugs [57], [58]. In our study, a combination treatment with melatonin and ER stress-inducing agents significantly increased p-PERK and p-eIF2α compared to treatment with or without melatonin only, downregulating translation and resulting in cell death.

In conclusion, our data suggest that inhibition of the PI3K/AKT/mTOR signaling pathway by melatonin combined with thapsigargin or tunicamycin efficiently attenuates growth and proliferation of B16F10 melanoma cells. The PI3K inhibitors LY294002 or wortmannin in combination with melatonin and thapsigargin/tunicamycin may enhance cell death.
